# Genome-Resolved Metaproteomic Analysis of Microbiota and Metabolic Pathways Involved in Taste Formation During Chinese Traditional Fish Sauce (Yu-lu) Fermentation

**DOI:** 10.3389/fnut.2022.851895

**Published:** 2022-04-07

**Authors:** Yueqi Wang, Yanyan Wu, Chunsheng Li, Yongqiang Zhao, Huan Xiang, Laihao Li, Xianqing Yang, Shengjun Chen, Leilei Sun, Bo Qi

**Affiliations:** ^1^Key Laboratory of Aquatic Product Processing, Ministry of Agriculture and Rural Affairs of the People’s Republic of China, National R&D Center for Aquatic Product Processing, South China Sea Fisheries Research Institute, Chinese Academy of Fishery Sciences, Guangzhou, China; ^2^Co-Innovation Center of Jiangsu Marine Bio-industry Technology, Jiangsu Ocean University, Lianyungang, China; ^3^Collaborative Innovation Center of Seafood Deep Processing, Dalian Polytechnic University, Dalian, China; ^4^College of Life Science, Yantai University, Yantai, China

**Keywords:** microbial enzymes, metaproteomics, fermentation, taste formation, fish sauce

## Abstract

Complex microbial metabolism is key to taste formation in high-quality fish sauce during fermentation. To guide quality supervision and targeted regulation, we analyzed the function of microbial flora during fermentation based on a previously developed metagenomic database. The abundance of most identified genes involved in metabolic functions showed an upward trend in abundance during fermentation. In total, 571 proteins extracted from fish sauce at different fermentation stages were identified. These proteins were mainly derived from *Halanaerobium*, *Psychrobacter*, *Photobacterium*, and *Tetragenococcus*. Functional annotation revealed 15 pathways related to amino acid metabolism, including alanine, aspartate, glutamate, and histidine metabolism; lysine degradation; and arginine biosynthesis. This study demonstrated the approaches to identify microbiota functions and metabolic pathways, thereby providing a theoretical basis for taste formation mechanisms during traditional fish sauce fermentation.

## Introduction

Traditional fish sauce is a widely used liquid condiment prepared from low-value aquatic products through natural fermentation for 10–18 months (1). It has been widely consumed for several centuries in several Asian countries, including South Korea, Japan, Thailand, and China. Typically, the natural fermentation method involving high-salt (25–30%) treatment is used to decompose and ferment biomolecules such as protein and lipids in fish under the cooperating action of microbial flora and endogenous enzymes (2, 3). The fermentation of fish sauce is key to its quality. During fermentation, microorganisms, enzymes, and metabolites form a complex system through a series of substance and energy exchanges. We previously found that the diversity of microbial communities significantly affects the qualitative characteristics of fermented fish sauce products and that profound changes occur in the metabolite and enzyme compositions that control the physical and chemical properties of the products (4). Microbial metabolism is responsible for the production of various major metabolites, such as organic acids and amino acids, as well as secondary metabolites (5, 6). These metabolites contribute to the nutritive value, taste, sensory properties, and unique flavor of fish sauce products. Extracellular enzymes produced by microorganisms play a key role in fermentation. Therefore, it is scientifically meaningful to analyze the microbial composition of fermented fish sauce and understand the role of the microbial communities and proteins involved.

With the rapid development of systems biology techniques, it has become possible to study the microbial community structure, functional genes, and functional proteins in fish sauce fermentation systems, assisted by high-throughput multi-omics technology. Metaproteomics is less restrictive for studying microbial populations, as labeling of identified proteins is not required. Moreover, the microbial functional gene dataset constructed via metagenomics can help identify protein types, obtain microbial sources, infer amino acid sequences, and track complex functions of the microbial population in the environment. Metaproteomics has been applied to study various fermented foods, such as soybean paste (7), Pu-erh tea (8), and rice wine (9). By assessing dynamic changes in proteins present in fermented products, metaproteomics provides information about the diversity of microorganisms, revealing information about the species involved, proteins expressed, and bacterial activity (10). The findings can help researchers better understand the role of microbial metabolism in food fermentation. However, it is difficult to construct a suitable sequence database for metaproteomic studies because of the complexity and heterogeneity of the microbial flora in fermented products (11). When using public databases, it may not be possible to identify proteins from poorly characterized microbial classifications. Therefore, a sample-specific metagenomic sequencing database will increase the protein identification rate, and the detectable database generated by tryptic peptide fragmentation will facilitate protein identification based on mass spectrometry.

In this study, we analyzed the function of microbial flora involved in traditional fish sauce fermentation based on the metagenomic database established in our previous study (4). Furthermore, metaproteomics was used to analyze the composition of microbial extracellular enzymes and microbial sources in the sauce at different fermentation stages. The data were used to construct a traditional fish sauce microbial extracellular enzyme database. We previously observed dynamic changes in taste compounds during fermentation (12), here, species annotations were carried out according to the metabolic pathways related to the formation of taste compounds and the coding genes of specific catalytic enzymes. This study predicted the roles of microorganisms across different metabolic pathways in the production of taste compounds were predicted based on the differences in their distribution. Moreover, this study will provide theoretical support for targeted process regulation and product quality monitoring of fish sauce, potentially promoting improvement of the traditional fish sauce industry.

## Materials and Methods

### Sample Preparation and Collection

#### Preparation of Aseptic Sampling Bottle

The cap of the sampling bottle was opened atop an aseptic ultra-clean workbench, and tweezers were used to pick an alcohol-soaked degreased cotton wad to wipe the inside as well as the surface of the bottle and the cap repeatedly, ensuring that the entire surface of the bottle was wiped with alcohol. The bottle and the bottle cap were placed on the ultra-clean workbench, and a UV lamp was turned on for sterilization and irradiation for 20 min. The bottle was closed tightly with the cap after the alcohol had evaporated; the bottle was then labeled with the location, date, and time of sample collection and other information.

#### Production Process of Traditional Fish Sauce

Fish sauce samples were collected from a processing factory located in Guangdong, China, as per a previously reported method (12). Anchovy (body length: 20 ± 3.0 cm; weight: 150 ± 30.0 g), collected from the South China Sea, were used as the raw material to produce the traditional fish sauce. After thawing, the whole anchovies were mixed with 20% salt and placed in a fermentation tank. Natural fermentation was conducted at 25°C for 1 year; the fermentation tank was stirred twice a day. The raw materials for fish sauce production and fermentation environments are shown in Supplementary Figure 1.

#### Collection Process of Fermentation Broth Samples

Traditional fermented fish sauce samples were collected from the top 10–12 cm of the upper surface of the fermentation broth at different fermentation stages. A total of five fermentation tanks were used for sampling in order to reduce sample error and ensure the stability of the experiment. The samples were collected after stirring. We collected 10.0 ± 0.2 L fermentation broth after 1, 3, 6, 9, and 12 months fermentation, respectively; hereafter, termed as 1M, 3M, 6M, 9M, and 12M, respectively. The sample replicates at each time point were well mixed on an ultraclean bench. The mixed samples collected at the same time point were defined as one sample. After collection, fermentation broth samples were filtered using qualitative filter paper (Cytiva Biotechnology, Hangzhou, China) and collected in aseptic sampling bottle. The filtrate was divided into three biological replicates and stored in a refrigerator at −80°C until further analysis.

### Metagenomic Sequencing Pre-processing

Metagenomic DNA was extracted using the OMEGA DNA kit (Omega Bio-tek Corp., Norcross, Georgia, United States) according to the manufacturer’s instructions. Then, the DNA quality then was assessed on 1% agarose gels. The DNA sample was fragmented to a size of 300 bp using the M220 nucleic acid shea (Covaris, MA, United States). A paired-end library was constructed using the TruSeq™ DNA sample prep kits (Illumina Co., San Diego, CA, United States). The libraries were sequenced on the Illumina Hiseq 2500 platform (13, 14).

### Quality Control and Gene Prediction

Low-quality and N-containing reads were removed to obtain clean data sequences for the subsequent analysis. The sickle program was then used to remove reads with lengths < 50 bp and an average quality <20. The optimized high-quality sequences were spliced and assembled using MEGAHIT, and contigs were obtained based on the overlapping relationship between k-mers. Open reading frames (ORFs) within contigs were predicted using MetaProdigal^1^, and nucleic acid sequences of genes longer than 60 bp were selected (15). The Least Common Ancestors algorithm was used to explore the non-redundant contigs of the nucleotide sequences in the NCBI nucleotide database. The relative abundance of unigenes was calculated by mapping to the number and length of unigenes (15).

### Protein Preparation and Digestion

The filtered sample (8 mL) was centrifuged at 15,000 × g for 5 min. After centrifugation, the supernatant was removed and purified by desalting on a PD-10 purified desalting column, and the effluent with protein components was collected after purification and desalting. The protein was precipitated by adding TAC/acetone solution (30 mL) for 12 h; the pellet obtained during the overnight precipitation was centrifuged at 10,000 rpm for 5 min to collect the protein precipitate, which was washed with acetone, resuspended in 8 M urea lysate, and sonicated (30% power, 4 s intervals per 2 s ultrasonic treatment, 10 cycles). After ultrasonication, the solution was centrifuged at 12,000 rpm for 10 min, and the supernatant was collected as a protein solution, 60 μL of which was collected in a centrifuge tube. Dithiothreitol solution (5 μL; 1 M) was added to this tube, and the mixture was shaken on a shaker and incubated at 37°C for 1 h in a water bath. Subsequently, iodoacetamide solution (20 μL; 1 M) was added to the mixture, which was then vortexed and allowed to stand at 25°C for 1 h under dark conditions. All processed protein samples were transferred to an ultrafiltration tube, and the supernatants were discarded after centrifugation. Thereafter, UA solution (100 μL; 8 M urea, 0.1 M Tris-HCl, pH 8.0) was added to the ultrafiltration tube, and the mixture was centrifuged; this step was repeated twice. Ammonium bicarbonate solution (100 μL, 0.05 M) was then added to the mixture, which was then centrifuged, and the supernatant was collected. This procedure was repeated three times. After replacing the collection tube, trypsin was added to the ultrafiltration tube at a protein:enzyme ratio of 50:1, followed by enzymatic hydrolysis at 37°C for 12–16 h (16).

### LC-MS/MS Analysis of Enzymatic Hydrolysates

To obtain quantitative results, the products were analyzed after trypsin digestion using liquid mass spectrometry, and three biological replicates of each sample were separated using an Easy nLC/Ultimate 3000 liquid phase system. The mobile phase buffer A was 0.1% formic acid, and buffer B comprised 80% acetonitrile and 0.08% formic acid. The sample was loaded through an autosampler at a flow rate of 600 nL/min to a 3 μm, 100 μm × 20 mm C18 mass spectrometer precolumn. It was separated using a 1.9 μm, 50 μm × 120 mm C18 column. The gradient elution parameters of the capillary high-performance liquid chromatography system are listed in Supplementary Table 1. Samples from different fermentation stages were separated using capillary high-performance liquid chromatography and analyzed using a Q-Exactive HF mass spectrometer, with set parameters as listed in Supplementary Table 2.

### Database Search and Protein Identification

Raw mass spectrometry data were obtained as RAW files, and the Mascot 2.1t (Matrix Science, London, United Kingdom) and Proteome Discoverer 1.4 (Thermo Fisher Scientific, Waltham, MA, United States) programs were used for library identification and quantitative analysis. A protein dataset of macrogen unigene translation of traditional fish sauce samples was used as the database. The RAW files were submitted to the Mascot server through Proteome Discoverer; the established metagenomic database of fish sauce was selected, and a database search was performed. The data are available from ProteomeXchange under identifier PXD031089.

### Bioinformatic Analysis

The FASTA protein sequences of differentially expressed proteins were analyzed using BLAST against the online eggNOG database^2^ and mapped to pathways in the Kyoto Encyclopedia of Genes and Genomes (KEGG) database.^3^ Enrichment analyses were performed based on Fisher’s exact test. Only functional categories and pathways with a *p* < 0.05 were considered significant. All analyses were performed with three biological replicates. Heat maps of hierarchical clustering were constructed using TBtools.^4^

## Results and Discussion

### Overview of Predicted Genes

The statistics of the spliced and assembled sequences during fish sauce fermentation are shown in Supplementary Table 3. In total, 268,885 contigs were obtained after assembly, and the average number of contigs was 126,159,706 bp. METAProdigal was used to make ORF predictions of the stitched contigs. After removing sequences <100 bp, 803,578 ORF sequences were obtained. The average number of ORF sequences per sample was 160,715. The ORF length distribution statistics are shown in Supplementary Table 3, with an average sequence length of 636 bp.

**FIGURE 1:**
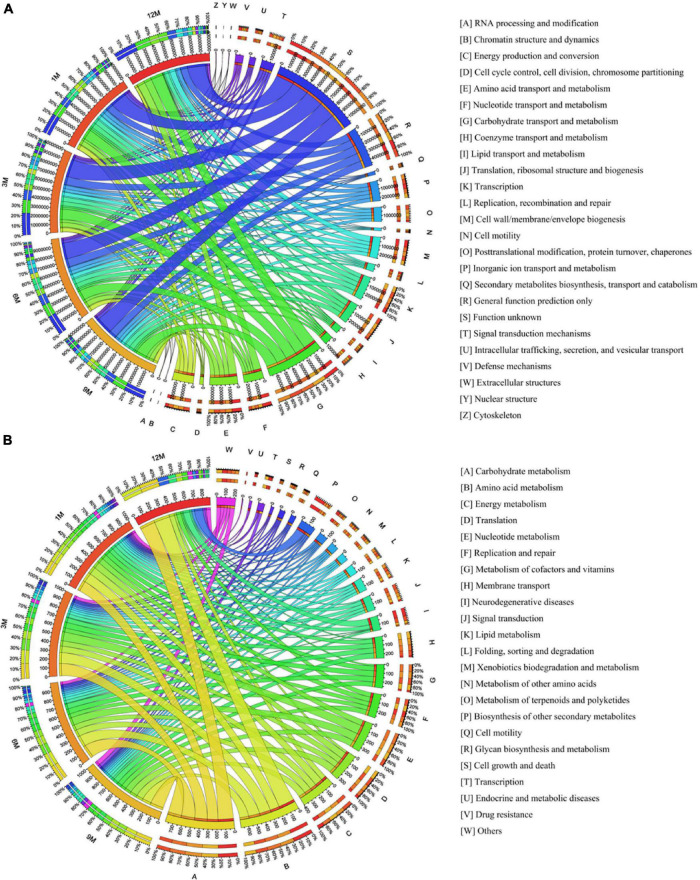
Functional annotation analysis of traditional fish sauce samples at different fermentation stages using eggNOG **(A)** and KEGG **(B)** databases, based on metagenomics.

Traditional fish sauce fermentation is conducted in an open fermentation environment containing many microorganisms that cannot be cultivated and have not been studied. Based on the eggNOG database, a functional annotation statistical analysis of fish sauce during fermentation was performed, and the results are shown in Figure 1A. Metabolism was the most direct functional classification related to the flavor of traditional fish sauce. Functional classifications related to amino acid metabolism and carbohydrate metabolism showed an upward trend, with average relative abundance of 6.68 and 13.70%, respectively. Based on KEGG database annotations, 39,899 genes were annotated during fish sauce fermentation. The abundance of genes associated with human disease-causing organisms showed a downward trend, consistent with the findings of our previous study on the microbial flora present during fish sauce fermentation. This result may be attributed to the growth of many salt-tolerant microorganisms at the later fermentation stage, which inhibited the growth of spoilage microorganisms in the fermentation system. Most identified genes categorized as metabolic functions showed an upward trend in abundance during fermentation (Figure 1B). At the initial fermentation stage, most genes were assigned to carbohydrate, amino acid, and energy metabolism. As fermentation progressed, amino acid, carbohydrate, and nucleotide metabolism gradually dominated the metabolic processes of the traditional fish sauce fermentation system, whereas the role of energy metabolism in the system gradually decline. Specifically, enhanced amino acid metabolism imparted the production of the characteristic umami and sweetness associated with fish sauce.

### Differentially Expressed Proteins and Bioinformatics Analysis

**FIGURE 2:**
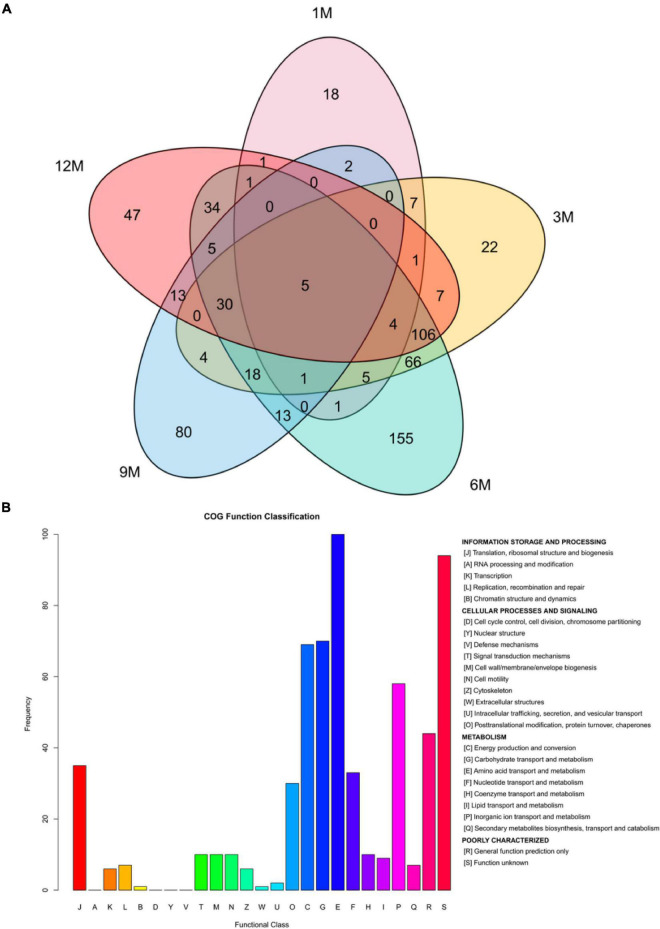
Venn plot analysis **(A)** and homologous classification **(B)** of proteins identified during the fermentation of traditional fish sauces.

To further determine the signal transduction and biochemical metabolic pathways involved in protein fermentation in traditional fish sauce, the difference in the proteome of the fish sauce was investigated using label-free MS/MS to explore the protein profiles related to taste compound formation. In total, 571 proteins were identified at different fermentation stages after the data were filtered at a false positive rate ≤ 0.01 (Figure 2A). During the traditional fish sauce fermentation, the 6M vs. 3M group shared the largest number of proteins, whereas the 1M vs. 9M group and 1M vs. 12M group shared a relatively small number of proteins. This result indicated that the protein composition in the traditional fish sauce fermentation system changed significantly with increasing fermentation time owing to the metabolism of the microorganisms. The changes in protein composition in the mid-fermentation system were the most obvious. Protein data derived from different fermentation stages, but with the same KEGG annotation results, were filtered (Supplementary Table 2). There were 36 types of enzymes involved in amino acid metabolism, including that of histidine ammonia-lyase, aspartate ammonia-lyase, alanine dehydrogenase, Glu/Leu/Phe/Val dehydrogenase, proline racemase, and dipeptidase. Functional annotation showed 15 pathways related to amino acid metabolism, including alanine, aspartate, glutamate, and histidine metabolism; lysine degradation; and arginine biosynthesis.

By analyzing the homologous classification of proteins, the 571 proteins identified were classified using eggNOG functional annotation (Figure 2B). Among them, the top-ranked functional classification was amino acid transport and metabolism (E), which was annotated to 98 proteins, followed by carbohydrate transport and metabolism (G), energy production and conversion (C), and organic ion transport and metabolism (P), which were annotated to 69, 69, and 57 proteins, respectively. These results showed that protein microbial activities during traditional fish sauce fermentation were mostly related to amino acid, carbohydrate, and energy metabolism. In addition, many proteins were involved in physiological processes, and most proteins in the functional category were involved in catalytic activity, whereas other proteins were involved in binding and transport activities. Translation, ribosomal structure, and biogenesis (J) and posttranslational modification, protein turnover, and chaperones (Q) were annotated to 35 and 30 proteins, respectively. The high-salt environment in fermented foods affects the metabolic mechanism of microorganisms, and high osmotic pressure causes water loss in microbial cells, leading to cell death (17). We speculated that in the traditional fish sauce fermentation system, microorganisms under high-salt stress would adjust their cellular ion concentration and produce enzymes to survive in a high osmotic pressure environment. Halophilic microorganisms mainly adapt to high osmotic pressure environments by adjusting the ion concentration in the cells and absorb compatible substances (such as sugars and amino acids) in the fermentation environment to balance the higher cellular ion concentration (18).

**FIGURE 3:**
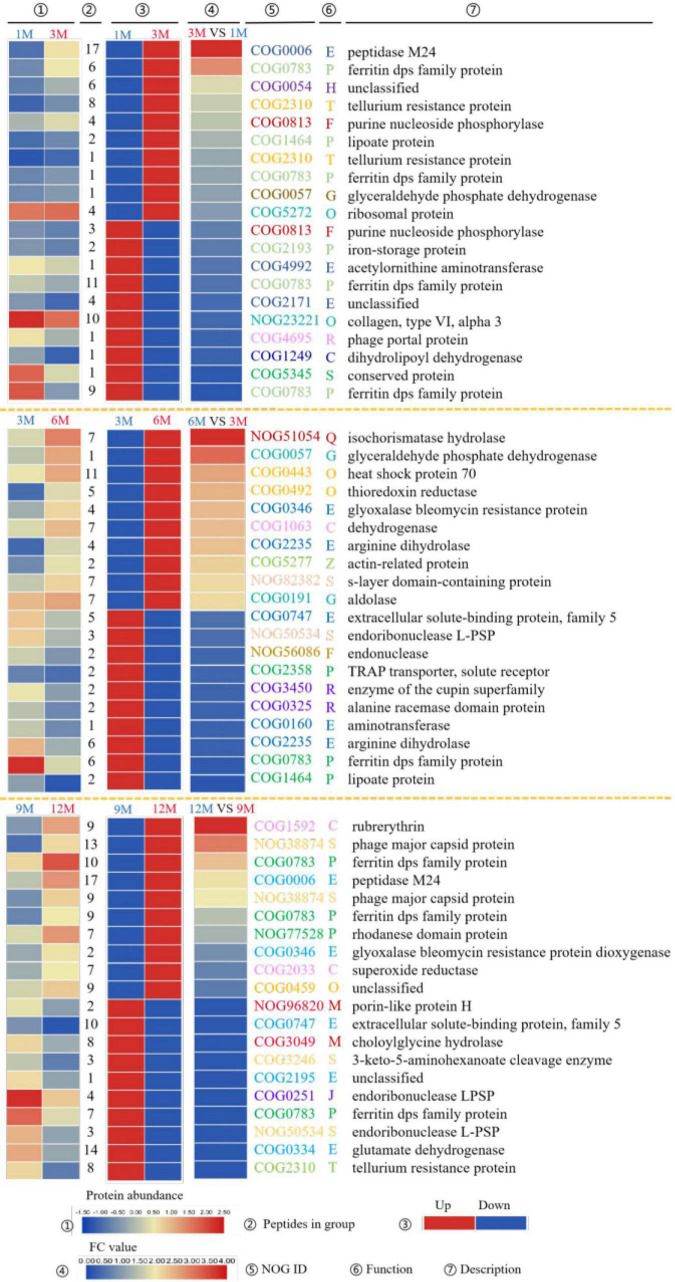
EggNOG annotations of differentially expressed proteins (DEPs) of traditional fish sauce based on metagenomics.

The top 10 most significantly upregulated or downregulated proteins in the 1M vs. 3M, 3M vs. 6M, and 9M vs. 12M groups are listed in Figure 3. Proteins with high mean proportion included peptidase M24 (COG0006), lipoate protein (COG1464), ferritin dps family protein (COG0783), and endoribonuclease L-PSP (COG0251). Peptidase is an enzyme capable of hydrolyzing peptide chains. Endopeptidases act on specific peptide bonds inside the polypeptide chain, and exopeptidases act on the N- or C-terminus of the polypeptide substrate, catalyzing the reaction. The hydrolysis of proteins and other substrates is the main biochemical process in traditional fish sauce fermentation systems. Peptidases cleave proteins in fermentation systems to release amino acids or polypeptides and increase the total nitrogen concentration in the soluble fraction (19). Protein degradation largely determines the quality and taste characteristics of traditional fish sauces. Duan et al. (20) found 11 peptidases in the flavor formation stage of shrimp paste based on metatranscriptome and 16S rRNA gene sequencing. In soy sauce fermentation, many enzymes related to amino acid metabolism from *Aspergillus oryzae* participate in the decomposition of these substances. Among them, dipeptidase, dipeptidyl aminopeptidase, and leucine aminopeptidase play positive roles in promoting the formation of flavor during soy sauce fermentation (21).

### Taxonomic Classification of the Identified Proteins

**FIGURE 4:**
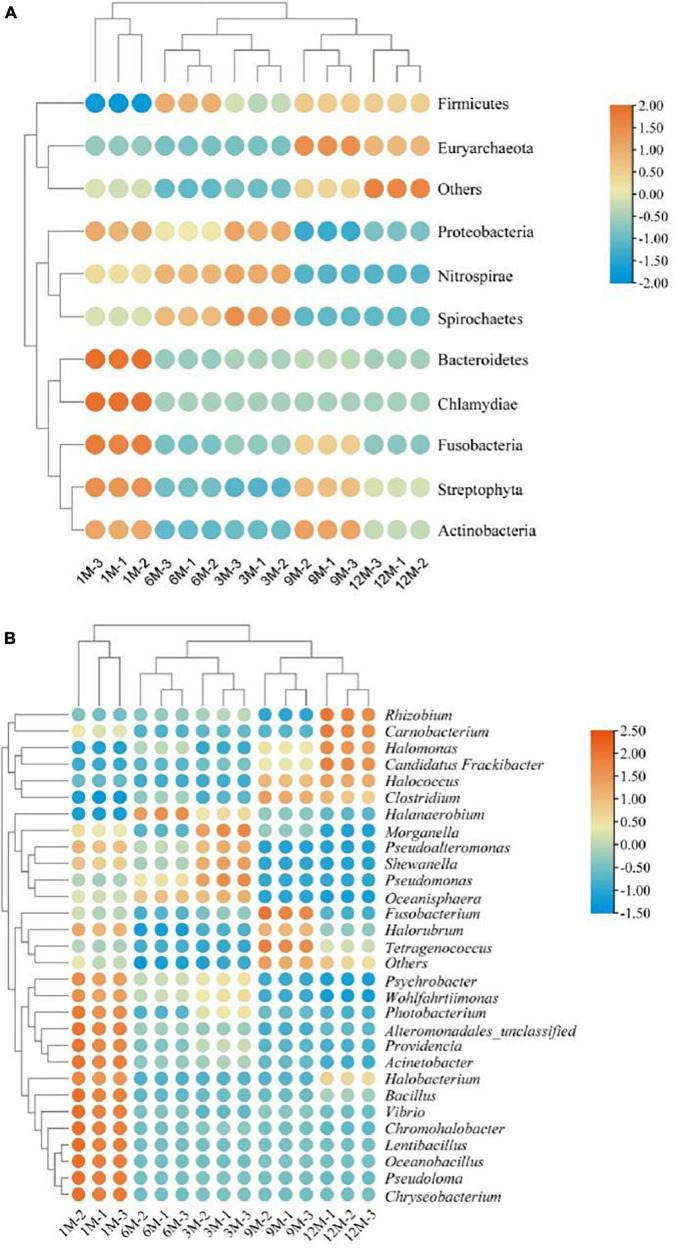
Protein-based taxonomic profiles of traditional fish sauce samples associated with fermentation time at the phylum level **(A)** and the genus level **(B)**.

In this study, we tracked the microbial sources of the identified proteins based on the constructed microbial functional gene dataset. Using Krona multi-layer interaction to visualize the protein species annotation information (Figure 4), the proteins in traditional fish sauce were found to be mainly derived from bacteria, accounting for 94% of the total protein. At the phylum level, there were 386 and 133 proteins derived from Firmicutes and Proteobacteria, accounting for 68 and 25% of the total protein, respectively. Ohshima et al. (22) have researched predominant bacterial communities of Thai fish sauce based on next-generation sequencing and demonstrated that Proteobacteria is the predominant bacterial phylum in the middle and late fermentation phases (22). Proteobacteria constitute the main microbial flora present in shrimp paste (23) and soybean paste (7); thus, Proteobacteria may play a key role in the quality characteristics of fermented foods.

At the genus level, proteins were mainly derived from *Halanaerobium*, *Psychrobacter*, *Photobacterium*, and *Tetragenococcus*, which are also common microbial sources of protein in fermented foods. *Halanaerobium* has been widely reported to be present in high-salt fermented foods, such as Korean traditional fermented fish sauce (24) and canned Swedish fermented herrings (25). *Tetragenococcus* can effectively improve the flavor and quality of fermented foods, and the intracellular aminopeptidase in *Tetragenococcus* has high proteolytic activity in high-salt environments (26). Therefore, *Tetragenococcus* is often used as a fermentation agent in the food industry. *Tetragenococcus* and *Halanaerobium* have similar stress regulation mechanisms under high-salt stress. Studies have shown that *Tetragenococcus* converts intracellular choline and carnitine into glycine betaine to adapt to high osmotic pressure environments (27, 28). Moreover, the microbial sources of proteins from other subclasses (Figure 4B) existed in fish sauce fermentation systems, such as *Photobacterium*, *Pseudomonas*, *Vibrio*, and *Shewanella*. As fish sauce is fermented in an open environment, it will inevitably be contaminated by external bacteria. However, considering that the number of bacteria is usually much lower under high-salt conditions, even very slight bacterial contamination could be distinguished by highly sensitive high-throughput sequencing. Therefore, these gene-expressing miscellaneous bacteria may have been introduced from the environment or raw materials but died or became dormant with the high growth of dominant bacteria during fermentation.

### Predicted Metabolic Pathways for Taste Compounds

**FIGURE 5:**
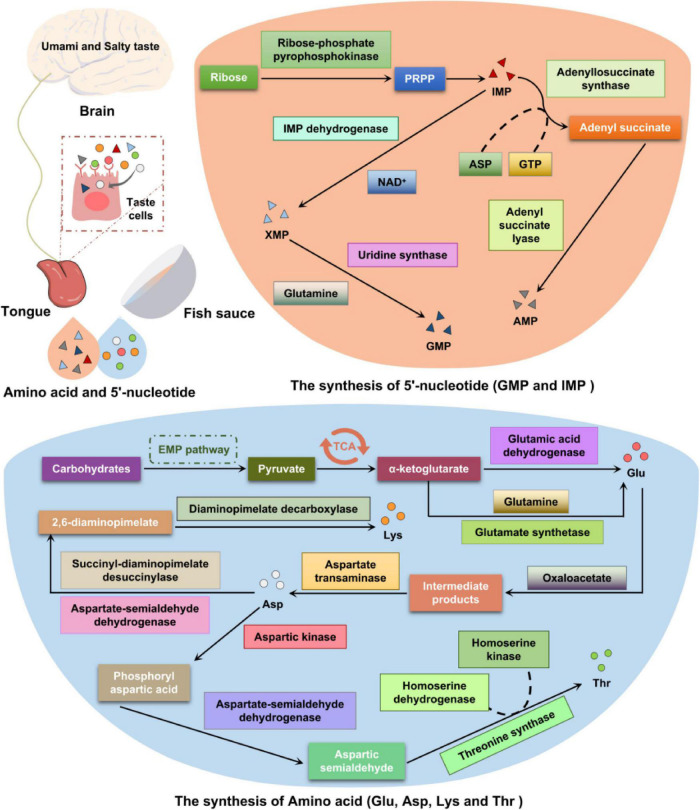
Summary of the metabolic pathways involved in the formation of taste compounds during the fermentation of traditional fish sauce.

Taste is the perception of water-soluble molecules in food by the human savory system. The overview of predicted metabolic pathways for taste compounds in fish sauce is shown in Figure 5. Taste buds composed of taste cells, which mainly exist on the surface and edge of the tongue, but are also distributed on the surface of the oral cavity and pharynx mucosa, act as taste receptors (29). Traditional fish sauce is widely used as a condiment because of its umami and salty taste. In our previous studies, we found the umami taste of fish sauce is produced during fermentation, and that the composition of taste compounds largely depends on the metabolism of microbial flora (12, 30). Umami is recognized as the fifth basic taste (along with salty, sweet, sour, and bitter) and denotes a pleasant savory or monosodium glutamate taste. In the traditional fish sauce fermentation system, amino acids and 5′-nucleotides dominate the production of its characteristic taste. The trend observed in free amino acids during fish sauce fermentation was as follows: concentration of fresh and sweet amino acids increased to 1345.44 and 899.17 mg/100 g, respectively, and concentration of bitter amino acids decreased to 885.32 mg/100 g after 12 months of fermentation. The contents of Glu, Asp, His, Leu, and Lys were significantly differed at different fermentation time points. The contents of Glu and Asp were the highest, accounting for about 35% of the total amino acid content after 12 months of fermentation (Supplementary Figure 2).

Taste-forming nucleotides play an important role in the freshness of aquatic products. Phosphate is formed on the 5′ carbon of the ribose to make the nucleotides taste umami. Typical representative nucleotides that are involved in taste formation during traditional fish sauce fermentation are 5′-guanosine monophosphate (5′-GMP) and 5′-inosine monophosphate (5′-IMP) (2, 30). Purine nucleotides are synthesized via *de novo* and salvage synthesis pathways (Supplementary Figures 3A,B), and the former is the main pathway (31). Purine and pyrimidine are important precursors of these two synthetic pathways, whereas glutamic acid and aspartic acid provide the necessary nitrogen (32). During fish sauce fermentation, the *de novo* synthesis of purine nucleotides can be divided into two stages (33). The first is 5′-IMP synthesis, which is catalyzed by ribose-phosphate pyrophosphokinase (EC: 2.7.6.1) and consumes ATP to form 5-phosphoribosyl-1-pyrophosphate (PRPP). Subsequently, PRPP is converted into 5′-IMP via 10 reaction steps, catalyzed by adenylosuccinate synthase (EC: 6.3.4.4), and aspartic acid provides amino acids for the synthesis of adenylosuccinate. Thereafter, adenylosuccinate removes fumaric acid under the action of adenylosuccinate lyase (EC: 4.3.2.2) to generate 5′-AMP. The production of 5′-GMP is also completed by a two-step reaction: (1) The precursor 5′-IMP is catalyzed by IMP dehydrogenase (EC: 1.1.1.205), using NAD + as a hydrogen acceptor to generate xanthosine monophosphate (5′-XMP); (2) 5′-XMP undergoes catalysis by uridine synthase (EC: 2.4.2.10 4.1.1.2) in a reaction with glutamine, which provides an amide group, to synthesize 5′-GMP (33, 34).

Amino acids are not only contributors of the nutrients and taste substances of traditional fermented foods but are also important precursors for flavor formation (35). Traditional fish sauce is a high-protein fermented food, and its flavor formation mainly comes from amino acid metabolism. However, the rate-limiting factor of flavor formation is not the release of free amino acids but the conversion of amino acids into flavor substances (36). Microbial proteases hydrolyze proteins in the raw materials during fish sauce fermentation and then catalyze the secondary proteolysis of amino acids to convert them into derivatives, generating taste compounds. The amino acid transamination reaction is a key step in the formation of flavor substances in fermented foods (37). Amino acid degradation and biosynthesis are dynamic equilibrium processes. The degradation of aromatic, branched-chain, and sulfur-containing amino acids must be conducted through transamination. The KEGG metabolic pathway map shows that the biosynthetic pathways of various amino acids are different, but the formation of their carbon skeletons is common, mainly from the citric acid cycle and pentose phosphate pathway. Based on the similarity of amino acid synthesis pathways, they can be divided into six categories, namely alanine, serine, glutamic acid, aspartic acid, histidine, and aromatic amino acid families (38). The biosynthetic pathways of representative umami and sweet amino acids are briefly described below.

The umami amino acid, glutamic acid, mainly converts carbohydrates to pyruvate through the EMP pathway; pyruvate is then converted to α-ketoglutarate through the TCA cycle (39). The biosynthetic pathway of glutamic acid is shown in Supplementary Figure 3C. α-Ketoglutarate, upon catalysis by glutamate synthetase (EC: 1.4.1.13, EC: 1.4.1.14), combines with the amine group of glutamine to generate glutamic acid. Alternatively, α-ketoglutarate can interact with free ammonia to produce glutamic acid under the action of glutamic acid dehydrogenase (EC: 1.4.1.3, EC: 1.4.1.4). Aspartic acid is mainly produced from the intermediate products of oxaloacetate and glutamic acid in the TCA cycle under the catalytic action of aspartate transaminase (EC: 2.6.1.1). The production of aspartic acid also involves L-asparaginase (EC: 3.5.1.1), aspartoacylase (EC: 3.5.1.15), and aspartate aminotransferase (EC: 2.6.1.1) during fish sauce fermentation. Moreover, aspartic acid is a synthetic precursor of threonine, lysine, and isoleucine (39, 40). The microbial proteases annotated to the glutamate metabolism pathway during fermentation were mainly derived from *Halanaerobium*, *Halococcus*, and *Halobacterium*.

The sweet amino acid lysine uses the umami amino acid aspartic acid as a precursor, and the biosynthetic pathway is shown in Supplementary Figure 3D. During traditional fish sauce fermentation, oxaloacetate forms a corresponding intermediate under a series of reactions, including phosphorylation, dehydrogenation, and reduction (40). The production of 2,6-diaminopimelate from aspartate is catalyzed by aspartate-semialdehyde dehydrogenase (EC: 1.2.1.11) and succinyl-diaminopimelate desuccinylase (EC: 3.5.1.18). Subsequently, 2,6-diaminopimelate is decarboxylated under the action of diaminopimelate decarboxylase (EC: 4.1.1.20) to form lysine. The microbial protease annotated to lysine biosynthesis during fermentation was mainly derived from *Halanaerobium*, *Psychrobacter*, *Vibrio*, and *Halococcus*. Another sweet amino acid, threonine, also uses aspartic acid as a precursor. Aspartic acid is catalyzed by aspartate kinase (EC: 2.7.2.4) to generate phosphoryl aspartic acid. Aspartate-semialdehyde dehydrogenase (EC: 1.2.1.11) reduces phosphoryl aspartic acid to aspartic semialdehyde and then generates threonine through the catalytic action of homoserine dehydrogenase (EC: 1.1.1.3), homoserine kinase (EC: 2.7.1.39), and threonine synthase (EC: 4.2.3.1). In addition, there are many other metabolic pathways involved in the production of threonine during the traditional fish sauce fermentation, and most enzymes in the metabolic pathway are covered in the annotation results.

## Conclusion

The current study sheds light on the understanding of microbiota and metabolic pathways involved in Chinese fish sauce fermentation. The results showed an upward trend in the abundance of most identified genes predicted to be involved in metabolic functions during fermentation. In total, 571 proteins extracted from fish sauce at different fermentation stages were identified after screening and filtering. The protein composition of the traditional fish sauce during fermentation changed significantly. The results demonstrated that the proteins in traditional fish sauce were derived from *Halanaerobium*, *Psychrobacter*, *Photobacterium*, and *Tetragenococcus* based on the constructed microbial functional gene dataset. Functional annotation showed 15 pathways related to amino acid metabolism, including alanine, aspartate, and glutamate metabolism, histidine metabolism, lysine degradation, and arginine biosynthesis. The composition of taste compounds largely depended on the metabolism of the microbial flora. The biosynthetic pathways of various amino acids were different, but the formation of their carbon skeletons, mainly via the citric acid cycle and the pentose phosphate pathway, was common. This study provided a detailed evaluation of fish sauce fermentation, enabling the development of better strategies for targeting regulation and quality monitoring of fish sauce.

## Data Availability Statement

The original contributions presented in the study are publicly available. This data can be found here: http://proteomecentral.proteomexchange.org/cgi/GetDataset?ID=PXD031089.

## Author Contributions

YWa: writing—original draft, conceptualization. YWu and LL: conceptualization. CL: writing—review and editing. YZ, XY, and SC: visualization. HX, LS, and BQ: software and formal analysis. All authors contributed to the article and approved the submitted version.

## Conflict of Interest

The authors declare that the research was conducted in the absence of any commercial or financial relationships that could be construed as a potential conflict of interest.

## Publisher’s Note

All claims expressed in this article are solely those of the authors and do not necessarily represent those of their affiliated organizations, or those of the publisher, the editors and the reviewers. Any product that may be evaluated in this article, or claim that may be made by its manufacturer, is not guaranteed or endorsed by the publisher.

## Footnotes

1
https://github.com/hyattpd/prodigal

2
http://eggnog.embl.de/

3
http://www.genome.jp/kegg/pathway.html

4
https://github.com/CJ-Chen/TBtools

## Abbreviations

E-nose: electronic nose
GC-IMS: gas chromatography–ion mobility spectrometry
GC-MS: chromatography-mass spectrometry
PCA: principal component analysis
VIP: variable importance in projection
VOC: volatile organic compounds.

## References

[1] Ding A Zhu M Qian X Shi L Huang H Xiong G Effect of fatty acids on the flavor formation of fish sauce. *LWT Food Sci Technol.* (2020) 134:110259. 10.1016/j.lwt.2020.110259

[2] Xu Y Zang J Regenstein JM Xia W. Technological roles of microorganisms in fish fermentation: a review. *Crit Rev Food Sci Nutr.* (2021) 61:1000–12. 10.1080/10408398.2020.1750342 32292041

[3] Chen Q Wang Y Wu Y Li L Yang X Chen S Investigation of fermentation–induced changes in the volatile compounds of *Trachinotus ovatus* (meixiangyu) based on molecular sensory and interpretable machine–learning techniques: comparison of different fermentation stages. *Food Res Int.* (2021) 150:110739. 10.1016/j.foodres.2021.110739 34865758

[4] Wang Y Li C Zhao Y Li L Yang X Wu Y Novel insight into the formation mechanism of volatile flavor in Chinese fish sauce (Yu-lu) based on molecular sensory and metagenomics analyses. *Food Chem.* (2020) 323:126839. 10.1016/j.foodchem.2020.126839 32334314

[5] Wang L Wang X Shi Z Shen L Zhang J Zhang J. Bovine milk exosomes attenuate the alteration of purine metabolism and energy status in IEC-6 cells induced by hydrogen peroxide. *Food Chem.* (2021) 350:129142. 10.1016/j.foodchem.2021.129142 33610842

[6] Zang J Xu Y Xia W Regenstein JM Yu D Yang F Correlations between microbiota succession and flavor formation during fermentation of Chinese low–salt fermented common carp (*Cyprinus carpio* l.) inoculated with mixed starter cultures. *Food Microbiol.* (2020) 90:103487. 10.1016/j.fm.2020.103487 32336353

[7] Xie M An F Yue X Liu Y Shi H Yang M Characterization and comparison of metaproteomes in traditional and commercial dajiang, a fermented soybean paste in northeast China. *Food Chem.* (2019) 301:125270. 10.1016/j.foodchem.2019.125270 31377619

[8] Zhao M An integrated metagenomics/metaproteomics investigation of the microbial communities and enzymes in solid-state fermentation of Pu-erh tea. *Sci Rep.* (2015) 5:10117. 10.1038/srep10117 25974221 PMC4431464

[9] Zhang B Kong L-Q Cao Y Xie G-F Guan Z-B Lu J. Metaproteomic characterisation of a Shaoxing rice wine “wheat Qu” extract. *Food Chem.* (2012) 134:387–91. 10.1016/j.foodchem.2012.02.057

[10] Wang Y Zhou Y Xiao X Zheng J Zhou H. Metaproteomics: a strategy to study the taxonomy and functionality of the gut microbiota. *J Proteom.* (2020). 219:103737. 10.1016/j.jprot.2020.103737 32198072

[11] Heyer R Schallert K Zoun R Becher B Saake G Benndorf D. Challenges and perspectives of metaproteomic data analysis. *J Biotechnol.* (2017) 261:24–36. 10.1016/j.jbiotec.2017.06.1201 28663049

[12] Wang Y Li C Li L Yang X Chen S Wu Y Application of UHPLC-Q/TOF-MS-based metabolomics in the evaluation of metabolites and taste quality of Chinese fish sauce (Yu-lu) during fermentation. *Food Chem.* (2019) 296:132–41. 10.1016/j.foodchem.2019.05.043 31202297

[13] Shen Y Wu Y Wang Y Li L Li C Zhao Y Contribution of autochthonous microbiota succession to flavor formation during Chinese fermented mandarin fish (*Siniperca chuatsi*). *Food Chem.* (2021) 348:129107. 10.1016/j.foodchem.2021.129107 33515949

[14] Yao Y Zhou X Hadiatullah H Zhang J Zhao G. Determination of microbial diversities and aroma characteristics of beitang shrimp paste. *Food Chem.* (2021) 344:128695. 10.1016/j.foodchem.2020.128695 33246688

[15] Escobar-Zepeda A Sanchez-Flores A Quirasco Baruch M. Metagenomic analysis of a Mexican ripened cheese reveals a unique complex microbiota. *Food Microbiol.* (2016) 57:116–27. 10.1016/j.fm.2016.02.004 27052710

[16] Xie M Wu J An F Yue X Tao D Wu R An integrated metagenomic/metaproteomic investigation of microbiota in dajiang-meju, a traditional fermented soybean product in Northeast China. *Food Res Int.* (2019) 115:414–24. 10.1016/j.foodres.2018.10.076 30599960

[17] Chen G Chen C Lei Z. Meta–omics insights in the microbial community profiling and functional characterization of fermented foods. *Trends Food Sci Technol.* (2017) 65:23–31. 10.1016/j.tifs.2017.05.002

[18] Chun BH Han DM Kim KH Jeong SE Park D Jeon CO. Genomic and metabolic features of *Tetragenococcus halophilus* as revealed by pan–genome and transcriptome analyses. *Food Microbiol.* (2019) 83:36–47. 10.1016/j.fm.2019.04.009 31202417

[19] Zhong Z Hu R Zhao J Liu W Kwok L-Y Sun Z Acetate kinase and peptidases are associated with the proteolytic activity of *Lactobacillus helveticus* isolated from fermented food. *Food Microbiol.* (2021) 94:103651. 10.1016/j.fm.2020.103651 33279076

[20] Duan S Hu X Li M Miao J Du J Wu R. Composition and metabolic activities of the bacterial community in shrimp sauce at the flavor–forming stage of fermentation as revealed by metatranscriptome and 16S rRNA gene sequencings. *J Agric Food Chem.* (2016) 64:2591–603. 10.1021/acs.jafc.5b05826 26978261

[21] Zhao G Ding LL Yao Y Cao Y Pan ZH Kong DH. Extracellular proteome analysis and flavor formation during soy sauce fermentation. *Front Microbiol.* (2018) 9:1872. 10.3389/fmicb.2018.01872 30158911 PMC6104182

[22] Ohshima C Takahashi H Insang S Phraephaisarn C Techaruvichit P Khumthong R Next–generation sequencing reveals predominant bacterial communities during fermentation of thai fish sauce in large manufacturing plants. *LWT Food Sci Technol.* (2019) 114:108375. 10.1016/j.lwt.2019.108375

[23] Lee SH Jung JY Jeon CO. Effects of temperature on microbial succession and metabolite change during saeu-jeot fermentation. *Food Microbiol.* (2014) 38:16–25. 10.1016/j.fm.2013.08.004 24290621

[24] Lee SH Jung JY Jeon CO. Bacterial community dynamics and metabolite changes in myeolchi-aekjeot, a Korean traditional fermented fish sauce, during fermentation. *Int J Food Microbiol.* (2015) 203:15–22. 10.1016/j.ijfoodmicro.2015.02.031 25770429

[25] Kobayashi T Kimura B Fujii T. Strictly anaerobic halophiles isolated from canned Swedish fermented herrings (Surströmming). *Int J Food Microbiol.* (2000) 54:81–9. 10746577 10.1016/s0168-1605(99)00172-5

[26] Kim KH Lee SH Chun BH Jeong SE Jeon CO. *Tetragenococcus halophilus* MJ4 as a starter culture for repressing biogenic amine (cadaverine) formation during saeu-jeot (salted shrimp) fermentation. *Food Microbiol.* (2019) 82:465–73. 10.1016/j.fm.2019.02.017 31027807

[27] Liu L Si L Meng X Luo L. Comparative transcriptomic analysis reveals novel genes and regulatory mechanisms of *Tetragenococcus halophilus* in response to salt stress. *J Indust Microbiol Biotechnol.* (2015) 42:601–16. 10.1007/s10295-014-1579-0 25563971

[28] Sugimoto S Saruwatari K Higashi C Tsuruno K Matsumoto S Nakayama J In vivo and in vitro complementation study comparing the function of dnaK chaperone systems from halophilic lactic acid bacterium *Tetragenococcus halophilus* and *Escherichia coli*. *Biosci Biotechnol Biochem.* (2008) 72:811–22. 10.1271/bbb.70691 18323638

[29] Liman ER Kinnamon SC. Sour taste: receptors, cells and circuits. *Curr Opin Physiol.* (2021) 20:8–15. 10.1016/j.cophys.2020.12.006 33709046 PMC7943026

[30] Wang W Zhou X Liu Y. Characterization and evaluation of umami taste: a review. *Trends Anal Chem.* (2020) 127:115876. 10.1016/j.trac.2020.115876

[31] Aird KM Zhang R. Nucleotide metabolism, oncogene–induced senescence and cancer. *Cancer Lett.* (2015) 356:204–10. 10.1016/j.canlet.2014.01.017 24486217 PMC4115046

[32] Sun L Zhang Z Xin G Sun B Bao XJ Wei Y Advances in umami taste and aroma of edible mushrooms. *Trends Food Sci Technol.* (2020) 96:176–87. 10.1016/j.tifs.2019.12.018

[33] Bhagavan NV Ha CE. Chapter 25 – nucleotide metabolism. 2nd ed. In: Bhagavan NV Ha C-E editors. *Essentials of Medical Biochemistry.* (San Diego, CA: Academic Press) (2015). p. 465–87.

[34] Wang Y Li F Chen J Sun Z Fu L. High–throughput sequencing–based characterization of the predominant microbial community associated with characteristic flavor formation in jinhua ham. *Food Microbiol.* (2021) 94:103643. 10.1016/j.fm.2020.103643 33279069

[35] Zhang L Yin M Zheng Y Xu C-H Tao N-P Wu X Brackish water improves the taste quality in meat of adult male *Eriocheir sinensis* during the postharvest temporary rearing. *Food Chem.* (2021) 343:128409. 10.1016/j.foodchem.2020.128409 33218856

[36] Ohmori T Mutaguchi Y Yoshikawa S Doi K Ohshima T. Amino acid components of lees in salmon fish sauce are tyrosine and phenylalanine. *J Biosci .Bioeng.* (2011) 112:256–8. 21658995 10.1016/j.jbiosc.2011.05.009

[37] Chua JY Liu SQ. Effect of single amino acid addition on growth kinetics and flavor modulation by *Torulaspora delbrueckii* in soy (tofu) whey alcoholic beverage fermentation. *Food Res Int.* (2020) 135:109283. 10.1016/j.foodres.2020.109283 32527478

[38] Kumar V Sharma A Kohli SK Yadav P Bali S Bakshi P Amino acids distribution in economical important plants: a review. *Biotechnol Res Innov.* (2019) 3:197–207.

[39] Zhang J Sun-Waterhouse D Su G Zhao M. New insight into umami receptor, umami/umami-enhancing peptides and their derivatives: a review. *Trends Food Sci Technol.* (2019) 88:429–38.

[40] D’Este M Alvarado-Morales M Angelidaki I. Amino acids production focusing on fermentation technologies – a review. *Biotechnol Adv.* (2018) 36:14–25. 10.1016/j.biotechadv.2017.09.001 28888551

