# Supplementary material for: Genome-Resolved Metaproteomic Analysis of Microbiota and Metabolic Pathways Involved in Taste Formation During Chinese Traditional Fish Sauce (Yu-lu) Fermentation
Source: Front Nutr. 2022 Apr 7;9:851895. doi: 10.3389/fnut.2022.851895 (PMC9021917; doi:10.3389/fnut.2022.851895)
Supplement: Supplementary file 1 [file Table_1.docx]

Table S1 The gradient elution parameters of the capillary high-performance liquid chromatography.

| Time (min) | A | B | Flow rate（nL/min） |
| --- | --- | --- | --- |
| 0 | 94% | 6% | 600 |
| 8 | 91% | 9% | 600 |
| 24 | 86% | 14% | 600 |
| 60 | 70% | 30% | 600 |
| 75 | 60% | 40% | 600 |
| 78 | 5% | 95% | 600 |
| 85 | 5% | 95% | 600 |
| 86 | 94% | 6% | 600 |
| 90 | 94% | 6% | 600 |
