# Supplementary material for: Genome-Resolved Metaproteomic Analysis of Microbiota and Metabolic Pathways Involved in Taste Formation During Chinese Traditional Fish Sauce (Yu-lu) Fermentation
Source: Front Nutr. 2022 Apr 7;9:851895. doi: 10.3389/fnut.2022.851895 (PMC9021917; doi:10.3389/fnut.2022.851895)
Supplement: Supplementary file 2 [file Table_2.docx]

Table S2 Parameter settings of Mass spectrometer instrument.

| Item | Full MS | dd-MS2/dd-SIM |
| --- | --- | --- |
| Microscans  Resolution | 1  120,000 | 1  15,000 |
| AGC target | 3.00E+06 | 5.00E+04 |
| Maximum IT | 80 ms | 45 ms |
| Scan range | 300-1400 m/z |  |
| NCE/stepped NCE |  | 27 |
| Spectrum data type | Profile | Centriod |
