# Supplementary figures and images for: Genome-Resolved Metaproteomic Analysis of Microbiota and Metabolic Pathways Involved in Taste Formation During Chinese Traditional Fish Sauce (Yu-lu) Fermentation
Source: Front Nutr. 2022 Apr 7;9:851895. doi: 10.3389/fnut.2022.851895 (PMC9021917; doi:10.3389/fnut.2022.851895)

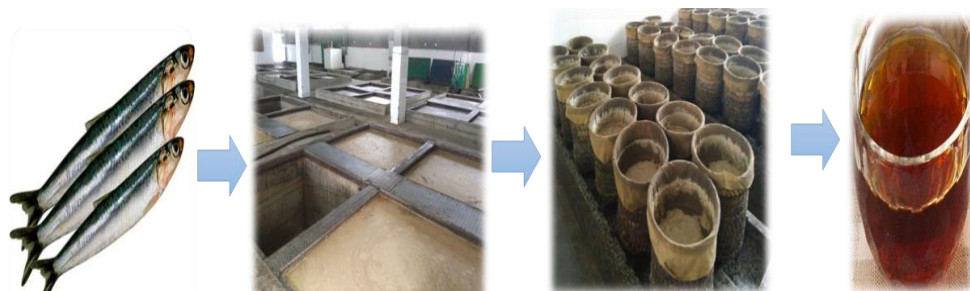

Fig.S1 Schematic diagram of fermentation environment in traditional fish sauce production.

Supplement: Supplementary file 3 [file Image_1.pdf]

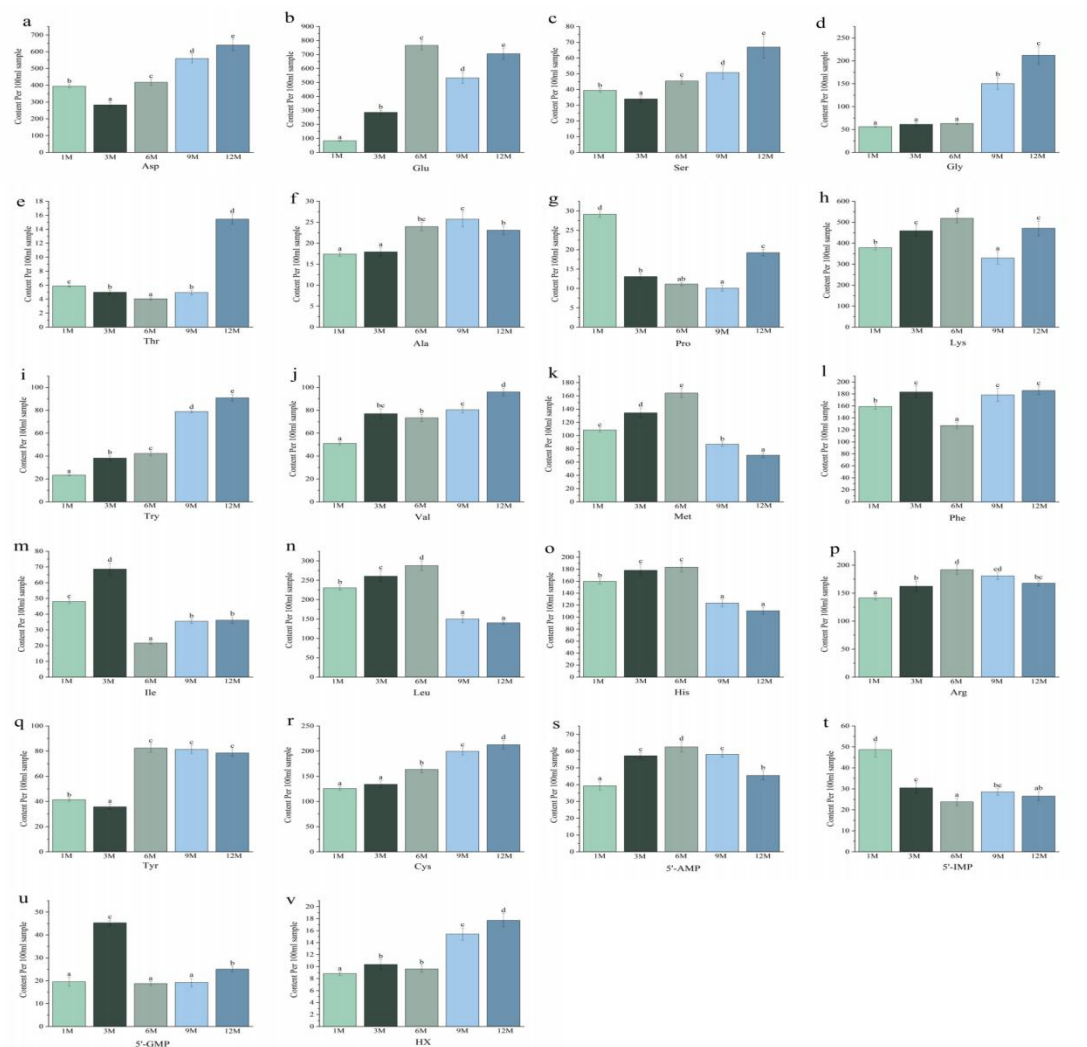

Fig.S2 Concentration of free amino acids and 5'-nucleotides in fish sauce during fermentation

Supplement: Supplementary file 4 [file Image_2.pdf]
