# Supplementary material for: Genome-Resolved Metaproteomic Analysis of Microbiota and Metabolic Pathways Involved in Taste Formation During Chinese Traditional Fish Sauce (Yu-lu) Fermentation
Source: Front Nutr. 2022 Apr 7;9:851895. doi: 10.3389/fnut.2022.851895 (PMC9021917; doi:10.3389/fnut.2022.851895)
Supplement: Supplementary file 5 [file Image_3.pdf]

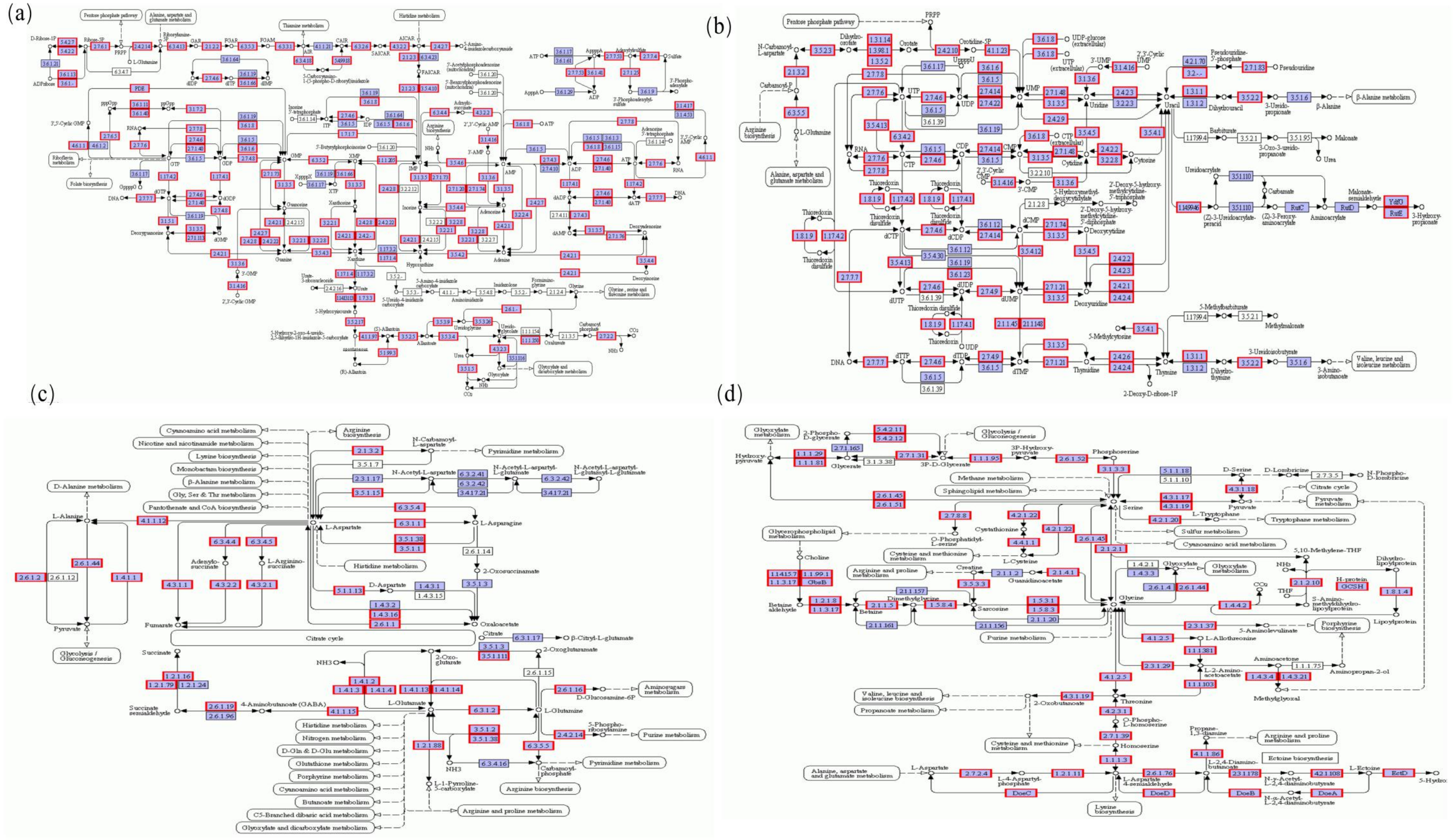

Fig. S3 Main metabolic pathways that form taste compounds during the fermentation of traditional fish sauce. Purine metabolic pathway (a), pyrimidine metabolic pathway (b), alanine, aspartate, and glutamate metabolic pathway (c), glycine, serine, and threonine metabolic pathway (d).
